# Identification of Peanut *Aux/IAA* Genes and Functional Prediction during Seed Development and Maturation

**DOI:** 10.3390/plants11040472

**Published:** 2022-02-09

**Authors:** Xiurong Zhang, Kun Zhang, Lu Luo, Yuying Lv, Yuying Li, Suqing Zhu, Bing Luo, Yongshan Wan, Xiansheng Zhang, Fengzhen Liu

**Affiliations:** 1 State Key Laboratory of Crop Biology, Shandong Key Laboratory of Crop Biology, Shandong Agricultural University, Tai’an 271018, China; zhangxr_1987@sina.com (X.Z.); kunzh@sdau.edu.cn (K.Z.); LuLuo0920@163.com (L.L.); lvyuying1990@163.com (Y.L.); 15610405658@163.com (Y.L.); zhusuqing0708@163.com (S.Z.); lubing_08@163.com (B.L.); 2 College of Agronomy, Shandong Agricultural University, Tai’an 271018, China; 3 College of Life Sciences, Shandong Agricultural University, Tai’an 271018, China

**Keywords:** auxin, *Aux/IAA*, peanut, seed development, growth period

## Abstract

Auxin-responsive genes *AUX/IAA* are important during plant growth and development, but there are few relevant reports in peanut. In this study, 44 *AhIAA* genes were identified from cultivated peanut, of which 31 genes were expressed in seed at varying degrees. *AhIAA-3A*, *AhIAA-16A* and *AhIAA-15B* were up-regulated, while *AhIAA-11A*, *AhIAA-5B* and *AhIAA-14B* were down-regulated with seed development and maturation. The expression patterns of seven genes, *AhIAA-1A*, *AhIAA-4A*, *AhIAA-10A*, *AhIAA-20A*, *AhIAA-1B*, *AhIAA-4B* and *AhIAA-19B*, were consistent with the change trend of auxin, and expression in late-maturing variety LM was significantly higher than that in early-maturing EM. Furthermore, allelic polymorphism analysis of *AhIAA-1A* and *AhIAA-1B*, which were specifically expressed in seeds, showed that three SNP loci in 3′UTR of *AhIAA-1A* could effectively distinguish the EM- and LM- type germplasm, providing a basis for breeding markers development. Our results offered a comprehensive understanding of *Aux/IAA* genes in peanut and provided valuable clues for further investigation of the auxin signal transduction pathway and auxin regulation mechanism in peanut.

## 1. Introduction

As one of the important plant hormones, auxin can not only participate in all aspects of plant growth and development, such as embryo development, apical dominance, organogenesis and morphogenesis, cell division and tissue differentiation, tropism, fruit ripening and organs aging, etc., but also be involved in the response to various abiotic stresses, such as salt and drought stress and exogenous hormone treatment [1,2,3,4,5,6,7]. Studies have also shown that auxin is the most important hormone that controls the ovary developing into fruit and causes parthenocarpy [8]. The regulated network of auxin involves complex processes such as regulation of gene transcription and protein expression, and according to the response degree, the regulated genes can be divided into primary response genes and secondary induced genes to auxin. The primary auxin-responsive genes mainly include *Auxin/indole-3-acetic acid* (*Aux/IAA*), *Small Auxin-up RNA* (*SAUR*) and *Gretchen Hagen 3* (*GH3*), which are always represented by a large multigene family in plants [5,7,9,10]. Among them, *Aux/IAA* is in relatively more research studies so far, and it is also a gene family with expression specificity in different tissues and developmental stages [7,11].

The *Aux/IAA* genes were isolated from soybean for the first time [12]. In *Arabidopsis thaliana*, 29 *Aux/IAA* genes were identified, which were found to distribute on five chromosomes [13]. In addition, studies on *Aux/IAA* genes and their function analysis have been reported in many other plants, such as cucumber and tomato, as well as in crops including wheat, rice and maize [1,3,11,14,15,16]. The *Aux/IAA* gene family encodes nuclear localization proteins with a short half-life period, and the Aux/IAA proteins are characterized by the presence of four highly conserved domains (domain I to IV) [17]. Domain I and domain II are located at the N-terminus, and their functions are inhibiting transcription and maintaining protein stability, respectively; domain III and domain IV are located at the C-terminus, which showed a very high homology with the auxin response factors (ARFs) [18,19,20]. With the development and wide application of molecular genetics technology, we are moving towards understanding the clear signal transduction pathway and feedback regulation mechanism of plant auxin.

With about 50% oil and 26% protein, peanut (*Arachis hypogaea* L.) is an important oil and economic crop that is grown worldwide with great nutritional value [21]. However, cultivated peanut is an allotetraploid (AABB, 2n = 4x = 40) with a total genome size of about 2.7 Gb, which is very large and complicated [22]. For a long time, research studies related to peanut genomes and functional genes have been relatively scarce due to the lack of genome information. In recent years, the released cultivated peanut genome data have provided strong support for the in-depth genetic research at the molecular and genetic level in peanut [22,23,24,25,26]. Previous studies on auxin in peanut mainly focused on dynamic changes in endogenous IAA content and distribution, and its influence on embryogenesis, but even such reports are limited [27,28,29]. In other plants, auxin has a strong effect on attracting and transporting nutrients, promoting fruit setting, assimilating transportation and dry matter accumulation in fruit and seed development. For example, in the grain filling stage of wheat, kernel growth and dry matter accumulation depend mainly on the concentration level of IAA and cytokinin (CTK) [30]. The exogenous auxin can also promote nutrient transportation, and auxin spraying to grain has an obvious effect on increasing yield in rice, and grain weight of cultivar with application of auxin under stress conditions also increased significantly [31]. Moreover, exogenous auxin treatment has a positive effect on increasing seed oil content in rapeseed [32]. Seed development and maturity are closely related to dry matter accumulation rate. In *Zoysia japonica*, IAA and gibberellin (GA) content slowly reduced with the seed becoming mature, while abscisic acid (ABA) content increased gradually, indicating that auxin plays an important role in the regulatory process of seed maturation [33]. In peanut, there are significant differences in growth period and seed development and maturation among varieties. Generally speaking, the early-maturing varieties usually have a faster seed development process, with a shorter growth period and earlier senescence of up-ground plants. On the contrary, the seed development process of late-maturing peanut varieties was relatively slow, with later senescence of above-ground plants. One of our previous studies also showed that seed fullness and dry matter accumulation rate were closely related to IAA content, and the peak value of IAA was significantly higher in late-maturing variety [29]. However, few studies were conducted at the molecular and genetic level in peanut. Only four possible *Aux/IAA* genes in peanut, named *PNIAA1*, *PNIAA2*, *PNIAA3* and *PNIAA4*, were identified, and the expression of *PNIAA3* was relatively higher in seeds [34], but no complete sequences and further research was reported.

In this study, we provide insight into a comprehensive understanding of *Aux/IAA* genes in peanut, and a total of 44 *AhIAA* genes were identified. The bioinformatics characters of these genes were analyzed, and expression differences analysis between early-maturing and late-maturing peanut varieties were further performed during the seed development process. Detailed sequence polymorphisms of the two genes specifically expressed in seeds were also analyzed among different germplasms, and the key SNP regulatory sites were predicted. The results will provide basic knowledge of *Aux/IAA* genes in peanut and provide valuable clues for further investigation of the auxin signal transduction pathway and auxin regulation mechanism in peanut.

## 2. Results

### 2.1. Identification of AhIAA Genes in Peanut

The hypothetical Aux/IAA protein sequences obtained by the Hidden Markov Model (HMM) analysis in peanut genome were submitted to websites NCBI-CDD, Pfam and SMART to confirm the Aux/IAA domain. Finally, a total of 44 sequences were confirmed to be peanut *Aux/IAA* genes and named as *AhIAA-1A*~*AhIAA-22A* and *AhIAA-1B*~*AhIAA-22B* based on their chromosomal locations. Detailed characteristics of *AhIAA* genes identified are listed in Table 1. The validated *AhIAA* genes and protein sequences are available in Supplementary Tables S1 and S2. Among the 44 AhIAA proteins, AhIAA-9A and AhIAA-9B were smallest with 133 aa, whereas AhIAA-15A was the largest with 455 aa. The molecular weight (MW) of protein ranged from 14.9 to 50.5 kDa, and the isoelectric point (pI) ranged from 4.86 (AhIAA-3B) to 9.13 (AhIAA-11A). The instability index (II) classified the proteins AhIAA-21A (36.24), AhIAA-8B (39.80), AhIAA-13B (37.92) and AhIAA-20B (36.98) as stable, which may be difficult to be degraded. Subcellular localization prediction showed peanut AhIAA proteins were located in nucleus and contained no transmembrane structure.

**Figure 1:**
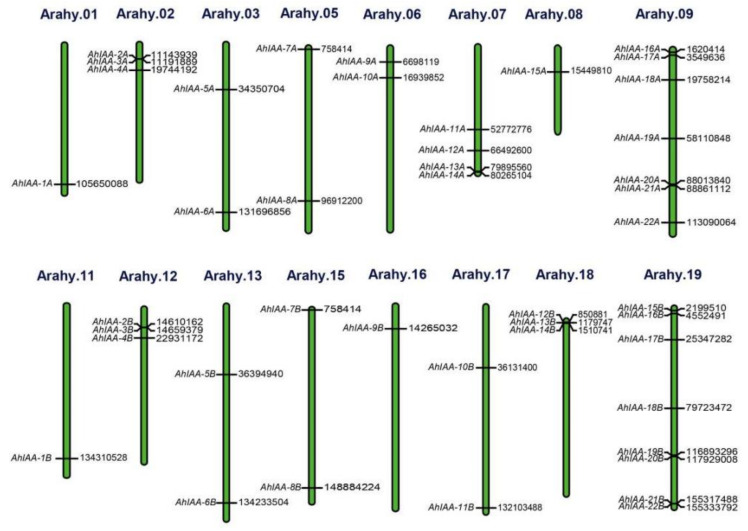
Chromosomal location of 44 *AhIAA* genes. “Arahy.*” represented chromosome number in cultivated peanut (https://peanutbase.org/gbrowse_peanut1.0, accessed on 10 January 2022). The name and corresponding start position of *AhIAA* genes were listed on the right and left side, respectively.

As shown in Figure 1, the 44 *AhIAA* genes were distributed on 16 peanut chromosomes unevenly. There were 22 genes, named as *AhIAA-1A*~*AhIAA-22A*, distributed on eight A- genome chromosomes including Arahy.01, Arahy.02, Arahy.03, Arahy.05, Arahy.06, Arahy.07, Arahy.08 and Arahy.09, and the other 22 genes were distributed on eight B- genome chromosomes including Arahy.11, Arahy.12, Arahy.13, Arahy.15, Arahy.16, Arahy.17, Arahy.18 and Arahy.19, named as *AhIAA-1B*~*AhIAA-22B*. On chromosomes Arahy.01, Arahy.08, Arahy.11 and Arahy.16, only one *AhIAA* gene was identified, respectively, whereas chromosome Arahy.19 (eight genes) had the most *AhIAA* genes, followed by chromosome Arahy.09 (seven genes).

The cultivated peanut is an allotetraploid (AABB), and homologous genes from the two subgenomes always share a very high sequence similarity. A total of 21 pairs of homologous *AhIAA* genes were identified through sequence alignment (Supplementary Table S3). The coding sequences alignment showed that *AhIAA-3A* and *AhIAA-3B* shared the lowest similarity of 58.14% due to a 327 bp deletion in *AhIAA-3B* compared with *AhIAA-3A*, while their remaining sequences had only two single base differences. The similarity of other pairwise homologous genes was all above 75%. Sequence similarities of 100% were found in homologous genes *AhIAA-7A* and *AhIAA-7B*, *AhIAA-9A* and *AhIAA-9B* and *AhIAA-14A* and *AhIAA-12B*, respectively, showing completely gene conservation between A and B subgenomes. The results of protein sequence alignment were very similar to those of coding sequence analysis (Table S3). The AhIAA-3B lacked a 109 aa fragment compared with AhIAA-3A, resulting in the lowest similarity of 57.85% between them. Although the gene *AhIAA-22A* and *AhIAA-22B* differed in the coding sequences with three single-base differences, the protein sequences encoded by them, AhIAA-22A and AhIAA-22B, were totally identical with a similarity of 100% (Table S3). Obviously, most pairwise homologous genes have similar chromosomal positions within the two subgenomes (Figure 1). For example, *AhIAA-1A* was located at 105 650 090 to 105 652 395 bp on chromosome Arahy.01, and its homologous *AhIAA-1B* was located at 134 310 530 to 134 312 921 bp on chromosome Arahy.11. However, there were some exceptions; for example, *AhIAA-11A*, *AhIAA-13A* and *AhIAA-14A* were located on chromosome Arahy.07, while their corresponding homologous genes *AhIAA-14B*, *AhIAA-13B* and *AhIAA-12B* were located on chromosome Arahy.18 (Figure 1, Table S3). Among all *AhIAA* genes, no homologous gene was identified for *AhIAA-10A*, and it may be a specific gene to the A subgenome.

### 2.2. Bioinformatics Analysis of AhIAA Genes

**Figure 2:**
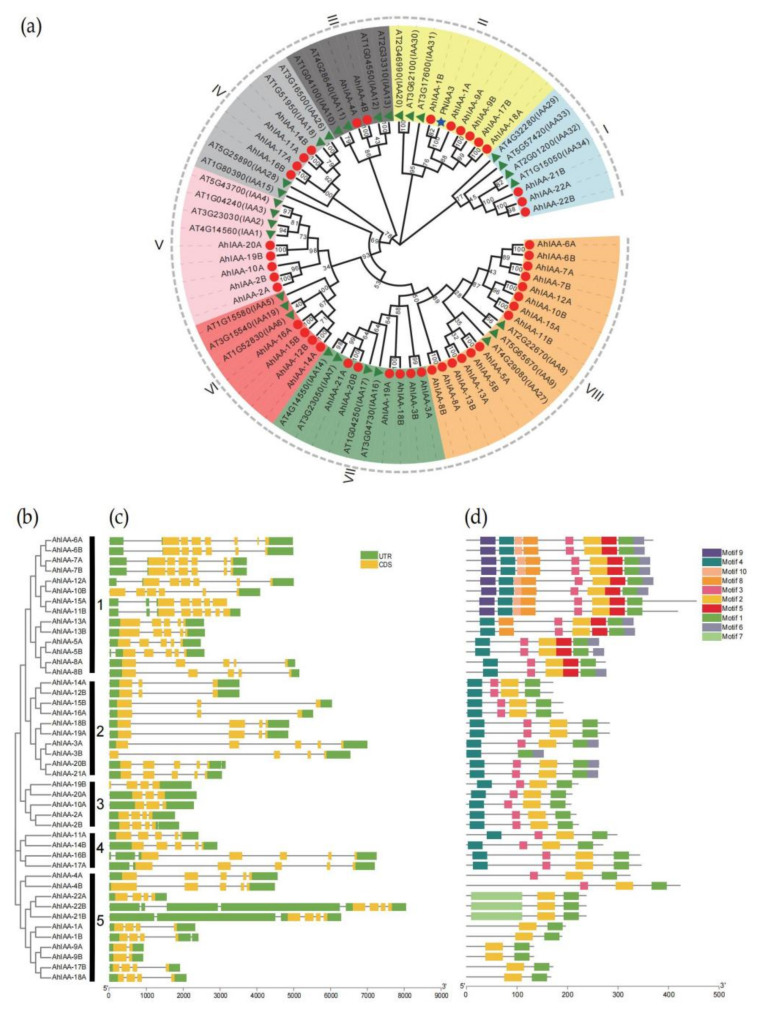
(**a**) Phylogenetic tree of AUX/IAA proteins from *Arabidopsis* and peanut. The phylogenetic tree was constructed using the Neighbor-joining (N-J) method with 1000 bootstrap replications. The eight groups were distinguished in different colors. Sequences from *Arabidopsis thaliana*, peanut (our study) and PNIAA3 (previous study) were labeled with red circle, green triangle and blue star, respectively. (**b**) The N-J phylogenetic tree of AhIAA proteins. Five clusters were displayed as 1 to 5. (**c**) Gene structure of peanut *AhIAA* genes. Green boxes indicated 5′ and 3′UTR; yellow boxes indicated exons; grey lines indicated introns. The sequence length could be estimated by the bottom scale. (**d**) The motif composition of peanut AhIAA proteins. The 10 motifs were displayed in different colored boxes, and the length of proteins could be estimated by the bottom scale.

The sequences and gene function were well known in *Arabidopsis thaliana*, so 29 *IAA* protein sequences from *Arabidopsis thaliana*, 44 sequences from our study and one sequence of PNIAA3 from the previous report [34] were used to construct an unrooted phylogenetic tree (Figure 2a). The 74 AUX/IAA proteins were classified into eight groups (from I to VIII), and most of them were clustered in group VIII. Remarkably, AhIAA proteins were more closely related to those AUX/IAAs in the same group than to the other AhIAAs from peanut, indicating that IAA proteins shared a relatively high conservation between the same group across species. Genes in the same cluster may have a similar function. *PNIAA3*, identified to be expressed in peanut seeds at high levels, was clustered with *AhIAA-1A* and *AhIAA-1B*, speculating that they may function during seed development. The protein sequences of 44 peanut *AhIAA* genes could be classified into five major clusters, namely cluster 1 to 5 (Figure 2b), consistent with the results from Figure 2a. The classes I, II and III were gathered into cluster 5, while class VI and VII formed cluster 2. Based on intron–exon analysis, the exons number of 44 *AhIAA* genes ranged from two to eight. For example, 12 (27.3%) genes had 5 exons, 9 (20.5%) had 4 exons, and 7 (15.9%) had 7 exons. Both *AhIAA-9A* and *AhIAA-9B* had only two exons, while *AhIAA-6A*, *AhIAA-11B* and *AhIAA-22B* had 8 exons (Figure 2c). Among all genes, *AhIAA-21B* and *AhIAA-22B* had the longest 5′UTR. We also used the MEME web server to search the conserved motifs which were shared with the AhIAA proteins. A total of 10 distinct conserved motifs were detected, and motif 1 and motif 2 were common in all AhIAA proteins, while motif 3 and motif 4 were not detected in most proteins in cluster 5, and genes *AhIAA-4A* and *AhIAA-4B* lacked motif 4 only (Figure 2d). The motif 5, motif 8, motif 9 and motif 10 were unique to genes in cluster 1. Motif 10 was detected only in three genes, *AhIAA-22A*, *AhIAA-21B* and *AhIAA-22B*. Obviously, genes and proteins in the same cluster usually share a similar intron–exon formation and motif distribution, especially within the pairwise homologous genes, with some exceptions.

**Figure 3:**
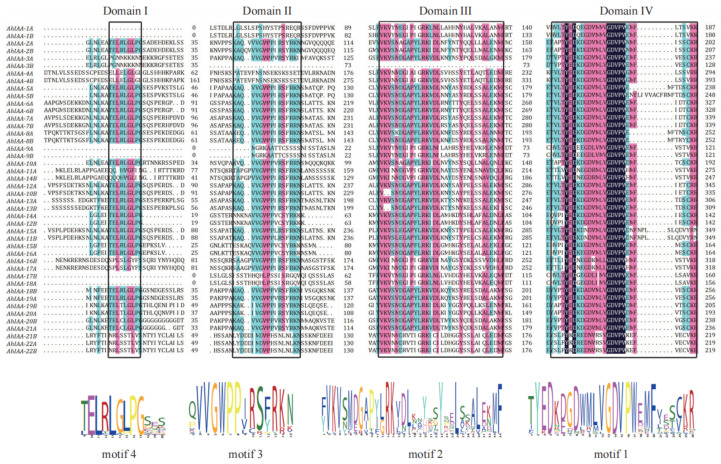
Conserved domains and detailed sequences information of their corresponding motifs detected in peanut AhIAA proteins.

Four conserved domains (domain I to IV) were also identified in most AhIAA proteins through sequences alignment (Figure 3), and motif 4, motif 3, motif 2 and motif 1 in Figure 2d were found to encode domains I to IV, respectively. These domains classified proteins to be Aux/IAAs [17]. Domain I was located at the N-terminus and showed a relatively higher variation, while six genes *AhIAA-1A*, *AhIAA-1B*, *AhIAA-9A*, *AhIAA-9B*, *AhIAA-18A* and *AhIAA-17B* lacked domain I. Aux/IAA proteins degradation was closely related to domain II [35], but AhIAA-3B lacked domain II, and may affect its stability and degradation. Domain III and IV, similar to ARF proteins [4,6,7], were located at the C-terminus, and both of them showed very high conservation among all AhIAA proteins (except AhIAA-3B).

Based on the genomic sequence, we obtained the potential promoter sequences, which are 2000 bp in length, within the upstream of 5′UTR of 44 *AhIAA* genes. *Aux/IAA* belongs to the early auxin responsive genes, in addition to typical eukaryotic promoter elements including CAAT-box and TATA-box, and many auxin-responsive elements were found in their promoter region from cis-acting elements prediction (Supplementary Figure S1). In addition, most genes contained cis elements in response to GA, ABA, salicylic acid (SA) and methyl jasmonate (MeJA), strongly indicating that *AhIAA* must play a key role in the intersection and interaction of different hormonal signaling pathways.

### 2.3. Tissue-Specific Expression of Peanut AhIAA Genes

**Figure 4:**
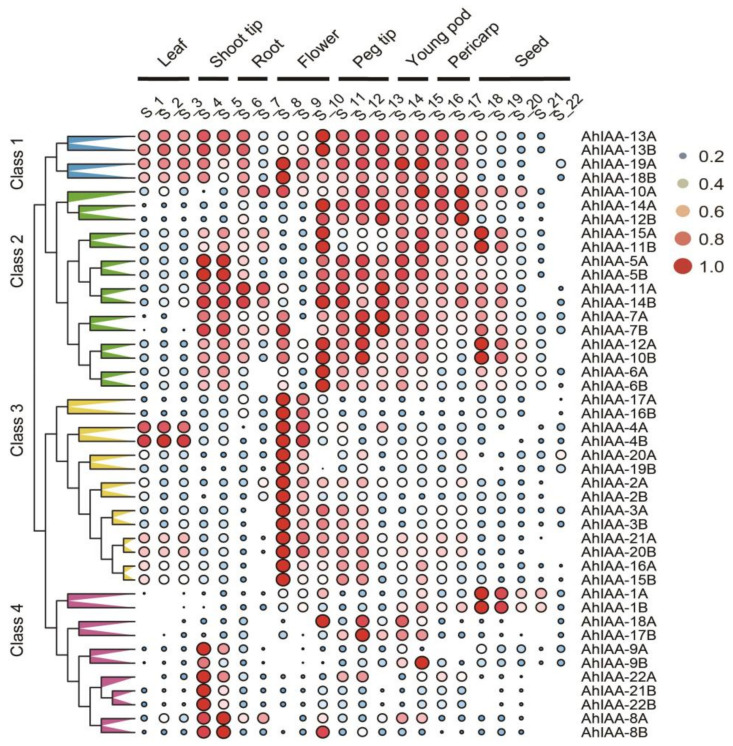
Heatmap of gene expression of *AhIAA* genes in different peanut tissue types. The 44 *AhIAA* genes were classified into four classes (class 1 to 4). The 22 tissues were renamed as S_1 to S_22. S_1, Seedling leaves; S_2, Main stem leaves; S_3, Lateral stem leaves; S_4, Vegetative shoot tip; S_5, Reproductive shoot tip; S_6, Roots; S_7, Nodule roots; S_8, Perianth; S_9, Gynoecium; S_10, Androecium; S_11, Aerial gynophore tip; S_12, Subterranean gynophore tip; S_13, Pattee 1 stalk; S_14, Pattee 1 pod; S_15, Pattee 3 pod; S_16, Pattee 5 pericarp; S_17, Pattee 6 pericarp; S_18, Pattee 5 seed; S_19, Pattee 6 seed; S_20, Pattee 7 seed; S_21, Pattee 8 seed; S_22, Pattee 10 seed. All FPKM values were normalized by log_2_ and scaled to 0-1 within each row.

Based on a published transcriptome dataset of 22 different tissue types in cultivated peanut [36], 44 *AhIAA* genes can be classified into four categories (Figure 4). Class 1 contained four genes, *AhIAA-13A*, *AhIAA-13B*, *AhIAA-19A* and *AhIAA-18B*, which had a relatively high gene expression level in most tissues except seeds. Class 2 consisted of 15 genes, and most of them were expressed at relatively higher levels in other tissues than leaves and seeds, while genes *AhIAA-14A* and *AhIAA-12B* were expressed at relatively lower level in shoot tips and root tissues, like that of *AhIAA-6A* and *AhIAA-6B* in the roots. Class 3 included 14 genes, and all of them had high gene expression in flowers; moreover, genes *AhIAA-4A* and *AhIAA-4B* were also expressed highly in leaves. The fourth class contained 11 genes, most of which had obvious gene expression only in shoot tips, while genes *AhIAA-1A* and *AhIAA-1B* were expressed in seeds at relatively higher level and *AhIAA-18A* and *AhIAA-17B* in the young pods with high expression. The above results indicated that *AhIAA* genes had tissue- and organ-expression specificity. Compared with other genes, *AhIAA-1A* and *AhIAA-1B* was specifically highly expressed in seeds, suggesting that they may play a more important role in seed development.

### 2.4. Differential Analysis of AhIAA Genes in Peanut Seeds between Early and Late- Maturing Varieties

**Figure 5:**
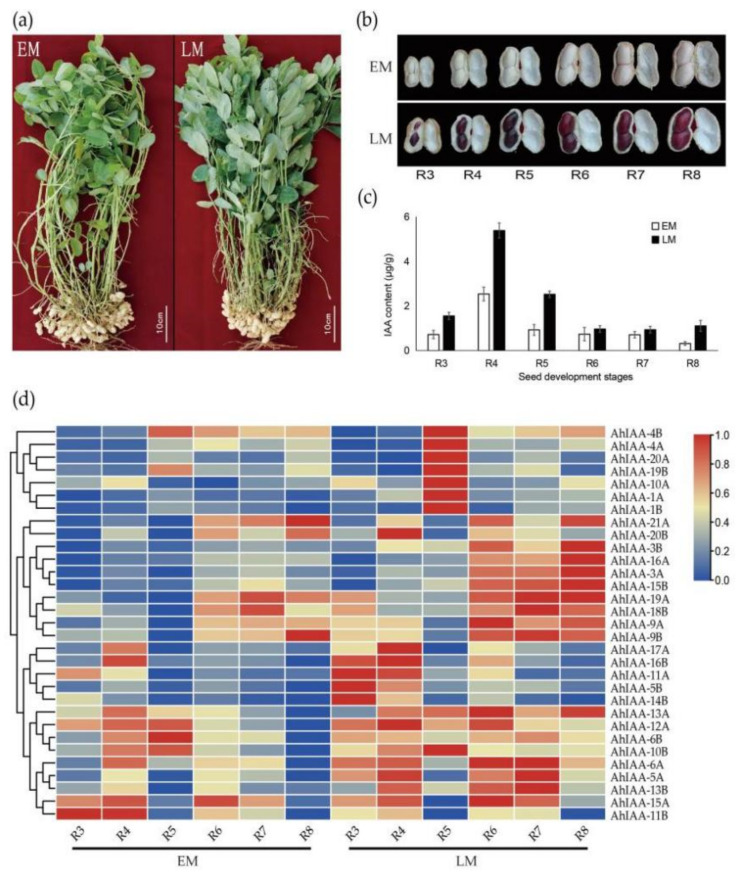
(**a**) The growth performance of EM and LM at R8 stage. EM has begun senescence and defoliation. EM and LM represented peanut varieties Fenghua2 and D666, respectively. (**b**) Seed development of EM and LM from R3 to R8. (**c**) IAA content differences between EM and LM during seed development process. (**d**) Heatmap of *AhIAA* gene expression in developing seeds of EM and LM. All expressions were normalized by log_2_ and scaled to 0-1 within each row.

The *AUX/IAA* genes were found to play important roles in fruit development and maturation [37,38], but there is no relevant research in peanut. In our study, an early-maturing variety Fenghua2 (EM) and a late-maturing variety D666 (LM) were used as materials (Figure 5a) for exploring the relationships of *AhIAA* genes and seed development and maturation. According to a previous report on pod development in peanut [39], we divided the pod developing period into nine stages, from R1 to R9. The R1 and R2 were the early stages of pod development, and seed development was nearly unable to be observed, while R9 was the late stage of pods over-maturing, and various contents in seeds had been stabilized accompanied by developmental cessation. Therefore, we selected six stages (R3 to R8) of the developing seed from two varieties (Figure 5b) for IAA content and *AhIAA* genes expression analysis.

The IAA content increased rapidly in the early stage of seed development, reached the maximum at the R4 stage, and then decreased sharply and maintained at a low level. The peak value in the early-maturing variety EM was significantly lower than that of the late-maturing variety LM (Figure 5c). The expression data of *AhIAA* genes were extracted from our transcriptomic dataset for differential analysis between the two varieties. Data analysis showed that 13 of the 44 peanut *AhIAA* genes were not expressed or were rarely expressed with FPKM <0.5 in seed, including *AhIAA-2A*, *AhIAA-7A*, *AhIAA-8A*, *AhIAA-14A*, *AhIAA-18A* and *AhIAA-22A*, and their homologous B subgenome genes *AhIAA-2B*, *AhIAA-7B*, *AhIAA-8B*, *AhIAA-12B*, *AhIAA-17B*, *AhIAA-21B* and *AhIAA -22B* (Supplementary Table S4). These 13 genes may not play major roles in seed development and maturation, and they were not involved in the subsequent analysis. From the expression patterns of 31 *AhIAA* genes in the developing seed (Figure 5d), seven genes’ expression was completely consistent with the IAA content changes, showing a trend of increasing at the early stage and then decreasing to a low level, including *AhIAA-1A*, *AhIAA-4A*, *AhIAA-10A*, *AhIAA-20A*, *AhIAA-1B*, *AhIAA-4B* and *AhIAA-19B*. It is speculated that these seven genes are more important in response to auxin during seed development. The expression of these seven genes reached the peak value at R5 stage, and expression in the LM variety was significantly higher than that in the EM variety, which was also similar to the IAA content difference. In addition, the expression of genes including *AhIAA-3A*, *AhIAA-16A* and *AhIAA-15B* showed an increasing trend with seeds becoming mature, while expression of another three genes including *AhIAA-11A*, *AhIAA-5B* and *AhIAA-14B* showed a decreasing trend with seed maturity, suggesting that these genes were also closely related to the seed developing process.

Combined with the tissue-specific results of gene expression (Figure 4), allelic polymorphism of *AhIAA-1A* and *AhIAA-1B* was further analyzed in LM and EM. Based on the results of simplified genome sequencing, three SNP loci were detected within 3′UTR of *AhIAA-1A*, and the position on chromosome Arahy.01 was 105652036 [A/G], 105652348 [T/-] and 105652355 [T/A]. However, no SNP locus was detected within *AhIAA-1B* between LM and EM. 

**Figure 6:**
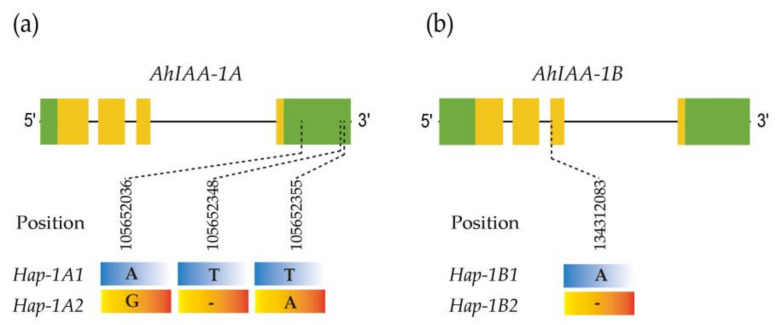
(**a**) Haplotypes of *AhIAA-1A*. Three SNP loci were located within 3′UTR, and their position were 105652036, 105652348 and 105652355 on chromosome Arahy.01. (**b**) Haplotypes of *AhIAA-1B*. One SNP were located within the third exon, and its position was 134312083 on chromosome Arahy.11. Green boxes indicated 5′ and 3′UTR, yellow boxes indicated exons, and grey lines indicated introns in gene structure.

Furthermore, we randomly selected 30 early-maturing and 30 late-maturing peanut germplasms, including landraces and breeding varieties (lines) (Supplementary Figure S2), based on the sequencing results, and SNP with a frequency of more than 5% was used for sequence diversity analysis (Supplementary Table S5). Three SNP loci detected within 3′UTR of *AhIAA-1A* formed two haplotypes *Hap-1A1* and *Hap-1A2* (Figure 6a), of which 34 germplasm were *Hap-1A1*, including 30 late-maturing germplasm and 4 early-maturing germplasm, while 26 *Hap-1A2* germplasm were the early-maturing type (Table 2, Figure S2). As to *AhIAA-1B* gene, only one SNP locus was detected, forming two haplotypes *Hap-1B1* and *Hap-1B2* (Figure 6b), including 56 and 4 germplasms, respectively, and 4 *Hap-1B2* germplasms were the late-maturing type (Table 2, Figure S2). All the germplasms can be divided into three haplotype-combination genotypes, namely *Hap-1A1/1B1*, *Hap-1A1/1B2* and *Hap-1A2/1B1*, including 30, 4 and 26 germplasms, respectively (Table 2, Figure S2). From the above results, it can be seen that haplotype *Hap-1A1* and *Hap-1A2* can effectively distinguish the germplasm maturity of the LM and EM types.

## 3. Discussion

Auxin is an important plant hormone, which is widely distributed and plays a vital role in plant growth and development. *Aux/IAA* is one of the most important primary auxin-response gene families, and its function should not be underestimated. A total of 29 *Aux/IAA* genes were isolated from *Arabidopsis*, which distributed on five chromosomes [13], and 31 *Aux/IAA* genes were isolated from rice [16], and 25 *Aux/IAA* genes were isolated from sorghum which was located on 9 chromosomes [40]. In this study, 44 *AhIAA* genes were identified from the peanut genome by bioinformatics methods, and they were located on 16 chromosomes (Table 1, Figure 1).

Cultivated peanut is an allotetraploid (AABB), and it is derived by the natural hybridization of the diploid wild species between A and B genomes [22]. Most of the homologous genes from the A and B subgenome usually have high sequence similarity [22,41]. Through sequence alignment analysis of peanut *AhIAA* genes, 21 pairs of homologous genes were identified between the A and B subgenome (Table S3). In phylogenetic evolutionary analysis, the paired homologous genes were preferentially clustered together, and sequence similarity of their coding and amino acid sequence were also very high; for example, the sequence similarity between pairwise *AhIAA-7A* and *AhIAA-7B*, *AhIAA-9A* and *AhIAA-9B* and *AhIAA-14A* and *AhIAA-12B* reached 100%, and similar gene structure and motif patterns were found among them, respectively (Table S3, Figure 2). The results also confirmed high conservation of this gene family during the evolution process [16,40]. However, gene sequences and expression levels of pairwise homologous genes were not always consistent (Figure 3, Figure 5, Figure S1). The results suggested that there may be differences in the regulation of homologous genes, and such differences probably caused a different contribution to the gene function. Further analysis of *AhIAA-1A* and *AhIAA-1B* showed *AhIAA-1B* is more conservative among the germplasm, while the variation of *AhIAA-1A* has a stronger correlation with maturity traits in peanut. Similar results had been reported on two homoeologous genes, *FAD2A* and *FAD2B*, encoding for the desaturase located on the A and B subgenome, respectively [41]. Some of our previous studies on the chloroplast Cu/Zn-SOD gene also showed consistent research findings [42].

Most of the isolated members of the Aux/IAA family are involved in the growth and development of roots [43,44], and a few reports showed that *Aux/IAA* genes influence fruiting; for example, silencing of the *SlIAA9* caused parthenocarpy, while silencing the *SlIAA27* gene not only caused parthenocarpy, but also changed the tomato size and shape [45]. Among the 44 *AhIAA* genes identified in our study, 13 genes were not expressed or were rarely expressed in seed from transcriptomic data analysis (Table S3), and the same result was obtained from gene expression in 22 different peanut tissue types (Figure 4), not excluding that these genes may play major roles in other tissues [7,11]. For example, *AhIAA-22A*, *AhIAA-21B* and *AhIAA-22B* were specifically expressed highly in shoot tips, while *AhIAA-14A* and *AhIAA-12B* were expressed relatively higher in flowers, young pods and pod pericarps (Figure 4). Among the remaining 31 *AhIAA* genes, seven of them, *AhIAA-1A*, *AhIAA-4A*, *AhIAA-10A*, *AhIAA-20A*, *AhIAA-1B*, *AhIAA-4B* and *AhIAA-19B*, had the highest expression in the middle stage of seed development, and the change trend was completely consistent with the auxin changes (Figure 5), indicating that they are key genes responding to auxin during seed development.

The maturing time varies greatly in peanut germplasm resources, and the longer growth period may provide conditions for seeds to accumulate more nutrients. The seed development process of early-maturing varieties are likely to be faster than that of late-maturing varieties. Therefore, peanut seed development and maturation is closely related with growth period, and maturing time is also a very important breeding character in peanut. Due to its underground fruiting characteristics, the key to judging seed maturity in peanut lies in the maturity of above-ground plants. The seed development process of late-maturing peanut varieties was relatively slow, with later senescence of above-ground plants. On the contrary, the up-ground plants aged earlier in early-maturing varieties, with a shorter growth period and relatively faster seed development process. Previous studies have shown that auxin plays a very important role in the regulation of fruit and seed development and has a close relationship with the dry matter accumulation ability, seed fullness and maturation [29,30], and exogenous auxin can promote nutrient transportation, showing a positive effect on increasing yield and seed oil content [31,32]. In our study, late-maturing variety LM and early-maturing EM were used for exploring the relationships between *AhIAA* genes and seed development. The middle stages (R4-R6) are the rapid accumulation period for nutrients and dry matter during peanut seed development, and seven *AhIAA* genes showed significantly higher expression in LM than that of EM (Figure 5). Among them, *AhIAA-1A* and *AhIAA-1B* were specifically expressed in seeds, and we further analyzed their sequence polymorphism. Thirty early-maturing germplasms and 30 late-maturing germplasms were randomly selected. After sequence analysis, we found that three SNP within *AhIAA-1A* could effectively distinguish the two types (Figure 6, Table 2, Figure S2, Table S5). The results showed that *AhIAA-1A* was closely related to peanut seed development and maturation. Based on the results, we are now developing corresponding PCR markers, which are expected to be used in breeding research. However, seed development and maturation is a complex biological process, which is certainly not determined by a single gene. In addition to *AhIAA-3A*, *AhIAA-16A* and *AhIAA-15B* showed an increasing trend with seeds becoming mature, while *AhIAA-11A*, *AhIAA-5B* and *AhIAA-14B* showed a decreasing trend, which are also closely related to the process of seed development. In addition, a lot of regulatory genes must be involved (Figure S1).

As an important regulator of the auxin signal transduction pathway, the *Aux/IAA* gene family is involved in many processes of plant growth and development through auxin-mediated transcription regulation, and it is a key protein in the transduction of auxin signaling [6]. In recent years, more and more studies are focused on the functional research of *Aux/IAA* family members and great progress has been made in some plants. In this study, we established a preliminary understanding of peanut *AhIAA* genes, analyzed the expression pattern in developing seed, discussed the differences between the EM- and LM-type germplasm, and predicted the key genes related to seed development. The results laid a foundation for the follow-up mechanism analysis of *AhIAA* regulatory network in peanut growth and development.

## 4. Materials and Methods

### 4.1. Peanut Genome and Sequences Resources

The peanut genome data (BioProject PRJNA419393) for allotetraploid *Arachis hypogaea* cv. Tifrunner (AABB) were used as reference, and assembled sequences were also available on the website PeanutBase (https://www.peanutbase.org/, accessed on 5 December 2019) [23]. Twenty chromosomes were numbered as Arahy.01-Arahy.20, where Arahy.01-Arahy.10 represented the A subgenome, and Arahy.11-Arahy.20 represented the B subgenome [23]. Moreover, whole genome annotation including files of gene models, coding sequences and protein data were downloaded from the PeanutBase. Sequences of 29 *Aux/IAA* genes in *Arabidopsis thaliana* were obtained from NCBI (https://www.ncbi.nlm.nih.gov/, accessed on 8 December 2019). Part of the sequence of *PNIAA3* was available from a previous study [34].

### 4.2. Identification of Aux/IAA Genes in Peanut Genome

The HMM file corresponding to the Aux/IAA domain (PF02309) was downloaded from the Pfam database (https://pfam.xfam.org/, accessed on 10 March 2020) [46] and used it as the query (*p* <0.001) to search the peanut protein sequence data. All candidate genes that may contain Aux/IAA domain based on hmmsearch [47] results were submitted to websites NCBI-CDD (https://www.ncbi.nlm.nih.gov/cdd, accessed on 15 March 2020), Pfam and SMART (http://smart.embl-heidelberg.de/, accessed on 15 March 2020) to confirm the Aux/IAA domain. The assumed genes with bitscore lower than 80 and genes with more conserved domains other than Aux/IAA were manually excluded. All the non-redundant and high-confidence genes were assigned as peanut Aux/IAA genes (AhIAA). Finally, a total of 44 sequences were confirmed to be peanut AhIAA genes and they were renamed based on position on chromosomes (Table 1).

### 4.3. Sequence Analysis

The length of protein sequences (number of amino acids), MW, pI and II of each AhIAA protein was obtained from the online program ExPASy (http://web.expasy.org/protparam/, accessed on 10 January 2022) [48]. The prediction of signal peptides was performed with SignalP 3.0 server (http://www.cbs.dtu.dk/services/SignalP-3.0/, accessed on 10 January 2022) [49] and iPSORT ((http://ipsort.hgc.jp/, accessed on 10 January 2022) [50]. Sequence alignment and phylogenetic analysis was conducted by MEGA V7.0 using the neighbor-joining (N-J) method with bootstrap replications of 1000 [51]. The exon–intron organization of peanut AhIAA genes was determined using the online program Gene Structure Display Server (GSDS: http://gsds.cbi.pku.edu.cn, accessed on 10 January 2022) [52] by comparing predicted coding sequences with their corresponding full-length sequences. The conserved motifs in the identified peanut AhIAA proteins were detected by the online program MEME (http://meme.nbcr.net/meme/intro.html, accessed on 10 January 2022) [53] with the maximum number of motifs of 10. The 2000 bp upstream regions of each AhIAA genes were taken as potential promoter sequences. The online softwares Place [54] (http://www.dna.affrc.go.jp/PLACE/, accessed on 10 January 2022) and Plantcare [55] (http://bioinformatics.psb.ugent.be/webtools/plantcare/html/, accessed on 10 January 2022) were used to predict cis-acting elements within promoter sequences. The software TBtools [56] was used to combine phylogenetic tree and others features figures. The charts of chromosomal location were produced by software MapChart [57].

### 4.4. Plant Materials and Sampling

An early-maturing variety Fenghua2 and a late-maturing variety D666 both developed by our lab were used as experimental materials, renamed as EM and LM, respectively (Figure 5a). Moreover, landraces or breeding varieties (lines) including 30 EM-type (EM_1 to EM_30) and 30 LM-type (LM_1 to LM_30) were also used for maturing phenotype and genotype identification (Figure S2). All the materials were grown in the test field of Agricultural Experiment Station of Shandong Agricultural University (36.15° N, 117.15° E), Tai’an, China. Developing seeds without seed coat at different stages (R3 to R8) of EM and LM were sampled for IAA content and transcriptome RNA-sequencing (RNA-seq) analysis. The fresh leaves were sampled for DNA extraction and genotype identification. All the collected samples were frozen in liquid nitrogen rapidly and stored in an −80 °C refrigerator before DNA or RNA extraction. After 110 days of sowing, the growth performance of the germplasm was photographed.

### 4.5. IAA Content Determination

According to\previous report [29], a high-performance liquid chromatography (HPLC) method was used to determine the IAA content. The chromatographic conditions were as follows: symmetry C18 column (150 mm × 4.6 mm, 5 μm), mobile phase of methanol and acetic acid aqueous solution (5‰ acetic acid), a flow rate of 0.9 mL/min, a column temperature of 25 °C, an injection volume of 15 μL, and a detection wavelength of 254 nm.

### 4.6. Data Analysis

Transcriptome RNA sequencing of developing seeds (R3 to R8) of EM and LM and simplified genomic resequencing of peanut germplasm were carried out in Biomarker Technologies Co., Ltd. The transcript abundance of peanut AhIAA genes was calculated as FPKM (fragments per kilobase of exon model per million mapped reads), and the average of three replicates was taken in analysis. The genes with FPKM values <0.5 were not selected for the following analysis [58]. Moreover, a published transcriptome dataset (BioProject PRJNA291488) of 22 different tissue types that represent the full development of cultivated peanut was obtained from the website PeanutBase (https://www.peanutbase.org/, accessed on 10 January 2022) [23]. The gene expression heatmap was created by TBtools [56] with standardized FPKM values by log2 and scaled to 0-1 within each row.

## 5. Conclusions

Auxin-responsive gene *AUX/IAA* has been widely studied in plants, but there are few relevant reports in peanut. In this study, 44 peanut *AhIAA* genes were identified by bioinformatics methods, of which 31 genes were expressed in seed at varying degrees. During seed development, the up-regulated genes *AhIAA-3A*, *AhIAA-16A* and *AhIAA-15B*, and down-regulated genes *AhIAA-11A*, *AhIAA-5B* and *AhIAA-14B* may be closely related to peanut seed development and maturation. Expression patterns of seven genes, *AhIAA-1A*, *AhIAA-4A*, *AhIAA-10A*, *AhIAA-20A*, *AhIAA-1B*, *AhIAA-4B* and *AhIAA-19B*, were consistent with the change trend of auxin, and gene expression level in LM was significantly higher than that in EM, so it was speculated that they were the key genes which responded to auxin and affected the seed maturation process. Furthermore, allelic polymorphisms of *AhIAA-1A* and *AhIAA-1B*, which were specifically expressed in peanut seeds, were analyzed among germplasms with different maturing times. The three SNP detected in 3′UTR of *AhIAA-1A* could effectively distinguish the EM- and LM-type germplasms, which may be the key regulatory sites for peanut maturity differences. We speculated that *AhIAA-1A* was closely related to peanut seed development and maturation. Furthermore, we are developing breeding markers based on the SNP loci of the gene *AhIAA-1A*. All the results provided a comprehensive understanding of *Aux/IAA* genes in peanut and provide valuable clues for further investigation of the auxin signal transduction pathway and the auxin regulation mechanism in peanut.

## Figures and Tables

**Table 1:** Features of *AhIAA* genes identified in peanut.

Name	Gene ID	ORF	Exon	aa	MW	pI	II
*AhIAA-1A*	arahy.Tifrunner.gnm1.ann1.IPD4BK.1	591	4	196	21.8	7.08	61.3
*AhIAA-2A*	arahy.Tifrunner.gnm1.ann1.PH74U7.1	654	4	217	24.6	5.4	59
*AhIAA-3A*	arahy.Tifrunner.gnm1.ann1.UBHF2S.1	786	5	261	29.3	6.09	45.05
*AhIAA-4A*	arahy.Tifrunner.gnm1.ann1.MDB4JZ.1	975	5	324	34.9	7.85	48.29
*AhIAA-5A*	arahy.Tifrunner.gnm1.ann1.85LRF7.1	789	5	262	28.4	6.75	48.32
*AhIAA-6A*	arahy.Tifrunner.gnm1.ann1.5EK85H.1	1110	8	369	39.4	6.04	44.37
*AhIAA-7A*	arahy.Tifrunner.gnm1.ann1.61CX9G.1	1092	7	363	38.6	7.53	46.5
*AhIAA-8A*	arahy.Tifrunner.gnm1.ann1.DW7R17.1	828	5	275	30.4	8.12	40.67
*AhIAA-9A*	arahy.Tifrunner.gnm1.ann1.QH3XYS.1	402	2	133	14.9	5.54	48.06
*AhIAA-10A*	arahy.Tifrunner.gnm1.ann1.43G8Y5.2	624	3	207	23.2	5.46	60.22
*AhIAA-11A*	arahy.Tifrunner.gnm1.ann1.F330XL.1	897	5	298	32.2	9.13	43.82
*AhIAA-12A*	arahy.Tifrunner.gnm1.ann1.XR81AR.1	1110	7	369	39.9	6.35	52.3
*AhIAA-13A*	arahy.Tifrunner.gnm1.ann1.N2VZ44.1	993	5	330	35.6	8.2	40.64
*AhIAA-14A*	arahy.Tifrunner.gnm1.ann1.0BZR9P.1	516	3	171	19.0	8.35	57.67
*AhIAA-15A*	arahy.Tifrunner.gnm1.ann1.FX782E.1	1368	7	455	50.5	8.26	46.25
*AhIAA-16A*	arahy.Tifrunner.gnm1.ann1.8QDS1I.1	576	3	191	21.8	6.76	45.54
*AhIAA-17A*	arahy.Tifrunner.gnm1.ann1.X1FQXP.1	1041	6	346	38.6	6.79	67.38
*AhIAA-18A*	arahy.Tifrunner.gnm1.ann1.AIW4ZZ.1	504	4	167	18.3	5.48	42.03
*AhIAA-19A*	arahy.Tifrunner.gnm1.ann1.PE1VQV.1	852	4	283	30.2	7.54	43.3
*AhIAA-20A*	arahy.Tifrunner.gnm1.ann1.5P5CS5.1	630	3	209	22.9	6.17	49.72
*AhIAA-21A*	arahy.Tifrunner.gnm1.ann1.QUY0YV.1	783	5	260	28.0	7.6	36.24
*AhIAA-22A*	arahy.Tifrunner.gnm1.ann1.6B28AS.1	714	4	237	27.0	5.11	49.76
*AhIAA-1B*	arahy.Tifrunner.gnm1.ann1.K6ZRIQ.1	570	5	189	21.1	7.82	54.82
*AhIAA-2B*	arahy.Tifrunner.gnm1.ann1.32M24S.1	669	5	222	25.0	5.17	59.32
*AhIAA-3B*	arahy.Tifrunner.gnm1.ann1.944YYF.1	459	4	152	17.4	4.86	48.5
*AhIAA-4B*	arahy.Tifrunner.gnm1.ann1.6LM78C.1	1272	5	423	46.1	8.61	50.76
*AhIAA-5B*	arahy.Tifrunner.gnm1.ann1.045PL1.1	819	6	272	29.5	6.75	48.22
*AhIAA-6B*	arahy.Tifrunner.gnm1.ann1.CY1TAH.1	1059	7	352	37.5	8.41	46.75
*AhIAA-7B*	arahy.Tifrunner.gnm1.ann1.1324HP.1	1092	7	363	38.6	7.53	46.5
*AhIAA-8B*	arahy.Tifrunner.gnm1.ann1.5PE8VQ.1	831	5	276	30.4	6.46	39.8
*AhIAA-9B*	arahy.Tifrunner.gnm1.ann1.WISN8Q.1	402	2	133	14.9	5.54	48.06
*AhIAA-10B*	arahy.Tifrunner.gnm1.ann1.XA7C26.1	1080	7	359	38.9	6.62	51.8
*AhIAA-11B*	arahy.Tifrunner.gnm1.ann1.P8YRGA.1	1257	8	418	46.2	6.65	50.3
*AhIAA-12B*	arahy.Tifrunner.gnm1.ann1.32PDNQ.1	516	3	171	19.0	8.35	57.67
*AhIAA-13B*	arahy.Tifrunner.gnm1.ann1.F753KD.1	1002	6	333	35.8	8.5	37.92
*AhIAA-14B*	arahy.Tifrunner.gnm1.ann1.5RC9P7.1	813	5	270	29.1	7.9	53.82
*AhIAA-15B*	arahy.Tifrunner.gnm1.ann1.KRC5M1.1	576	3	191	21.8	5.87	46.94
*AhIAA-16B*	arahy.Tifrunner.gnm1.ann1.QSK9AI.1	1032	7	343	38.3	6.98	68.12
*AhIAA-17B*	arahy.Tifrunner.gnm1.ann1.F8XE83.1	516	4	171	18.8	5.48	40.21
*AhIAA-18B*	arahy.Tifrunner.gnm1.ann1.Q1FV6H.1	852	4	283	30.3	7.6	43.86
*AhIAA-19B*	arahy.Tifrunner.gnm1.ann1.VW0TTK.1	666	4	221	24.3	5.56	52.8
*AhIAA-20B*	arahy.Tifrunner.gnm1.ann1.CB6084.1	789	6	262	28.1	7.6	36.98
*AhIAA-21B*	arahy.Tifrunner.gnm1.ann1.T6Y945.3	714	6	237	27.0	5.11	49.76
*AhIAA-22B*	arahy.Tifrunner.gnm1.ann1.M3JKN9.4	714	8	237	27.0	5.11	49.76

Gene ID, ID in cultivated peanut genome(https://peanutbase.org/gbrowse_peanut1.0, accessed on 10 January 2022); ORF, open reading frame; aa, amino acid; MW, molecular weight (kDa); pI, isoelectric point; II, instability index.

**Table 2:** The number of EM- and LM-type germplasms among each haplotype and haplotype combination of *AhIAA-1A* and *AhIAA-1B*.

Germplasm	*AhIAA-1A*		*AhIAA-1B*		*AhIAA-1A/AhIAA-1B*		
	*Hap-1A1*	*Hap-1A2*	*Hap-1B1*	*Hap-1B2*	*Hap-1A1/1B1*	*Hap-1A1/1B2*	*Hap-1A2/1B1*
EM-type	4	26	30	0	4	0	26
LM-type	30	0	26	4	26	4	0
Total	34	26	56	4	30	4	26

## Data Availability

Not applicable.

## Supplementary Materials

The following are available online at https://www.mdpi.com/article/10.3390/plants11040472/s1, Figure S1. Some of the predicted cis-acting elements in potential promoter sequence of *AhIAA* genes in peanut. Figure S2. Genotypes and growth performance of germplasm after 110 days of sowing. Table S1. The cDNA sequences of validated *AhIAA* genes in peanut. Table S2. The protein sequences of validated *AhIAA* genes in peanut. Table S3. The similarity of coding and protein sequences of pairwise homologous *AhIAA* genes. Table S4. The FPKM value of 44 *AhIAA* genes from transcriptome data in peanut. Table S5. Allele polymorphism analysis of *AhIAA-1A* and *AhIAA-1B* among early maturing and late-maturing peanut germplasms.
