# Supplementary figures and images for: Identification of Peanut Aux/IAA Genes and Functional Prediction during Seed Development and Maturation
Source: Plants (Basel). 2022 Feb 9;11(4):472. doi: 10.3390/plants11040472 (PMC8874715; doi:10.3390/plants11040472)

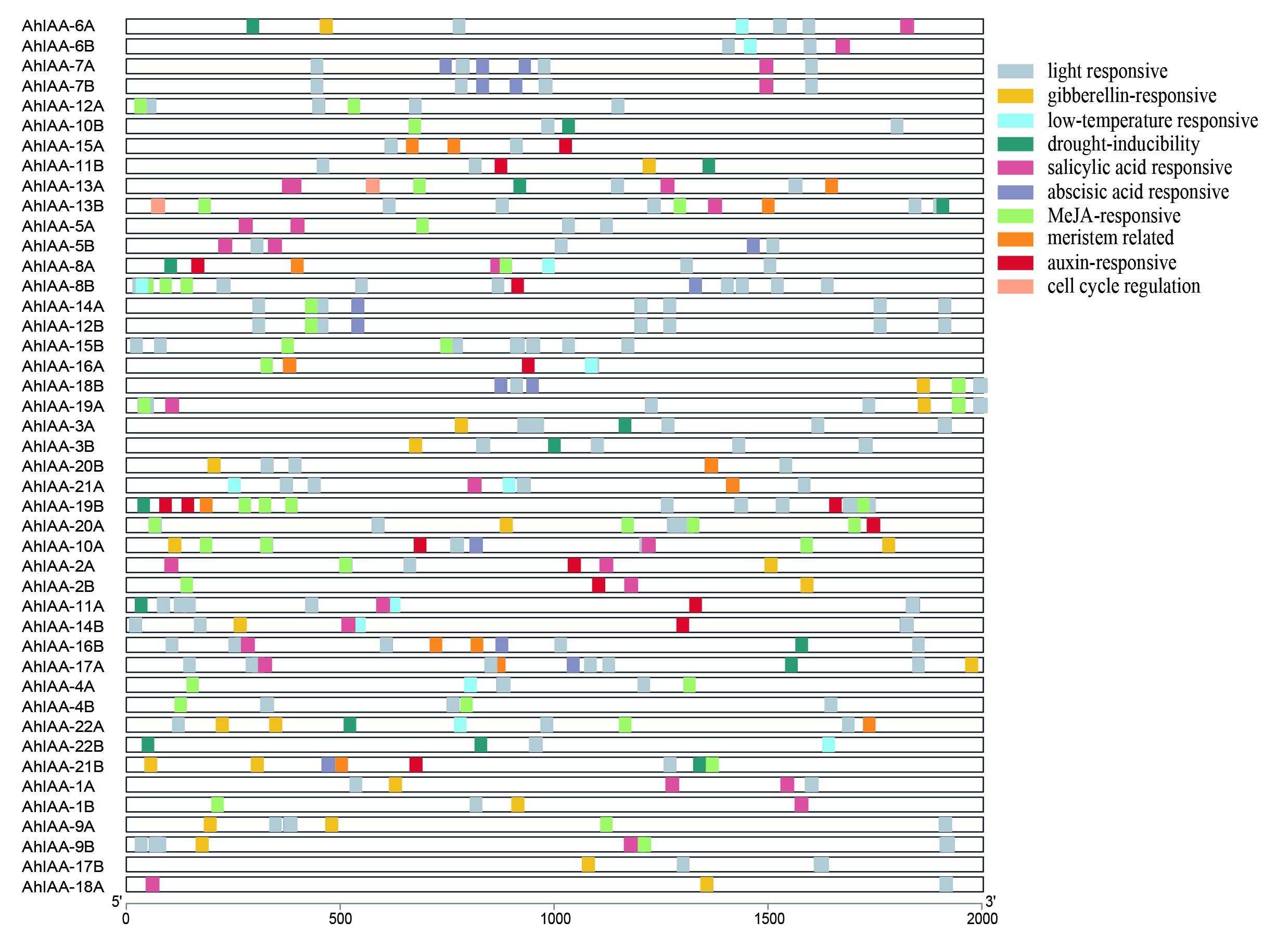

Supplement: Supplementary file 1 [file plants-11-00472-s001.zip › supplementary files/Figure S1.jpg]

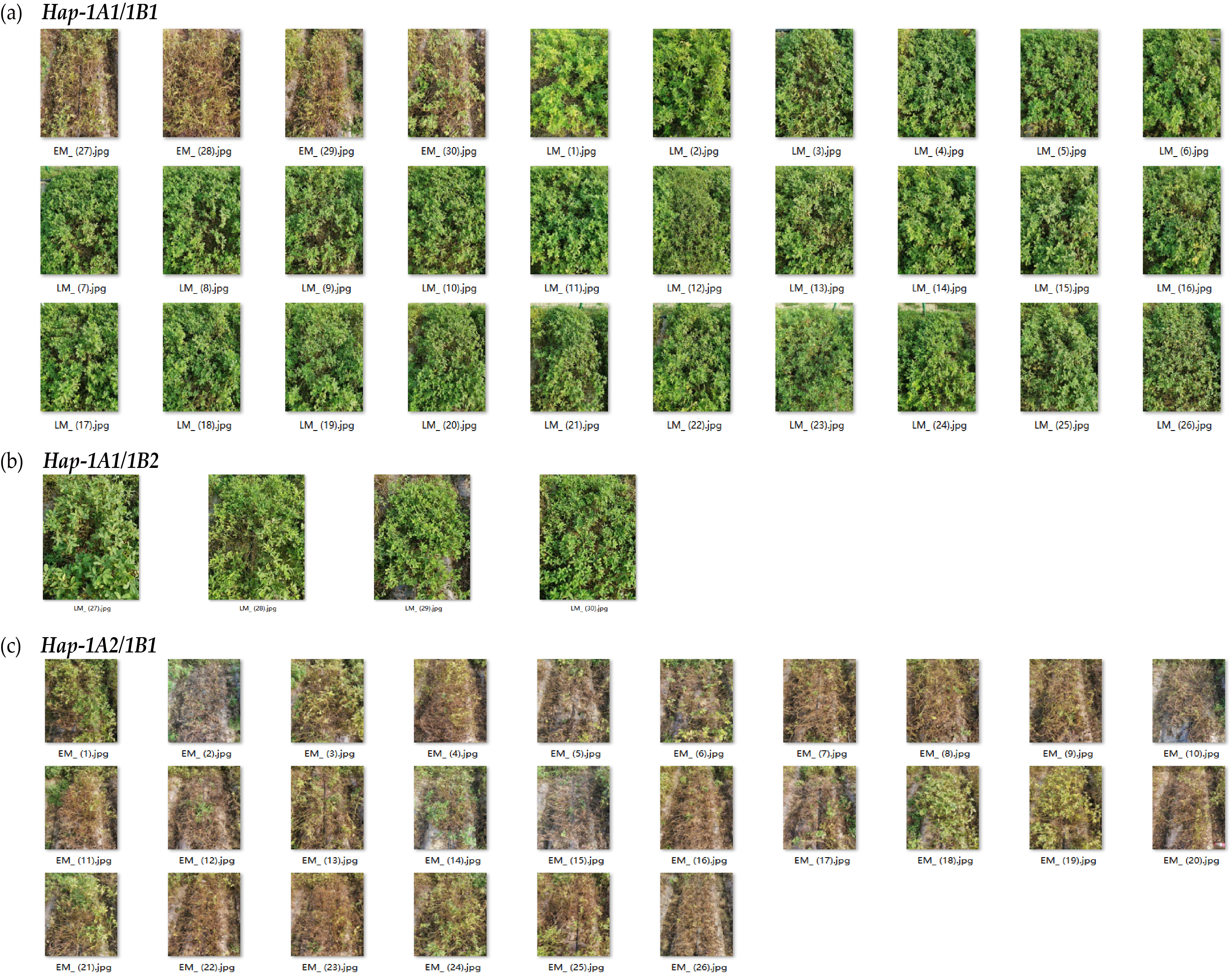

Supplement: Supplementary file 1 [file plants-11-00472-s001.zip › supplementary files/Figure S2.jpg]
